# Modulation of Ire1-Xbp1 Defense Pathway in Encephalomyocarditis Virus-Infected HeLa Cells

**DOI:** 10.3390/v17030360

**Published:** 2025-03-02

**Authors:** Anna Shishova, Yury Ivin, Ekaterina Gladneva, Ksenia Fominykh, Ilya Dyugay, Anatoly Gmyl

**Affiliations:** 1 Chumakov Federal Scientific Center for Research and Development of Immune-and-Biological Products of Russian Academy of Sciences (Institute of Poliomyelitis), 108819 Moscow, Russia; ivin_uu@chumakovs.su (Y.I.); gladneva_ee@chumakovs.su (E.G.); foxenia@gmail.com (K.F.); dyugay.ilya@gmail.com (I.D.); shishova_aa@chumakovs.su (A.G.); 2 Institute for Translational Medicine and Biotechnology, First Moscow State Medical University (Sechenov University), 117418 Moscow, Russia

**Keywords:** encephalomyocarditis virus, ER stress, UPR, IRE1, XBP1, unconventional splicing

## Abstract

A key contributor to the pathogenicity of viruses is their interaction with cellular defense mechanisms, including UPR (unfolded protein response) that counteracts the accumulation of misfolded proteins in the endoplasmic reticulum (known as ER stress). One of the UPR branches is mediated by the IRE1 (inositol-requiring enzyme 1) protein, which possesses protein kinase and RNase activities that facilitate the unconventional cytoplasmic splicing of XBP1 mRNA, leading to the upregulation of the XBP1 transcription factor. In this study, we demonstrate that Encephalomyocarditis Virus (*Cardiovirus rueckerti*) is able to suppress IRE1-dependent XBP1 activation. HeLa cells infection with EMCV resulted in the modulation of phosphorylated IRE1 levels throughout the infection cycle. Viral infection did not result in the accumulation of spliced XBP1 mRNA. Moreover, the addition of a chemical inducer of ER stress (dithiothreitol) to infected cells led to a markedly lower accumulation of spliced XBP1 mRNA as compared to the level of this mRNA in inducer-treated mock-infected cells. Thus, our results demonstrate the ability of picornaviruses to modulate another defensive activity of the host cell.

## 1. Introduction

Encephalomyocarditis virus (EMCV), a member of the *Cardiovirus* genus within the *Picornaviridae* family, is a non-enveloped virus characterized by its positive single-stranded RNA genome [1]. This zoonotic pathogen is found globally and has the potential to infect a diverse array of host species, including rodents, non-human primates, swine, and humans [2,3,4]. Thus, gaining insight into the specific interactions between EMCV and its host cells is essential for the effective treatment and management of this infection.

EMCV utilizes its security proteins, L and 2A, to modulate host cell functions and enhance viral replication within infected cells [5,6]. The L protein of EMCV, although devoid of intrinsic enzymatic activity, contains a CHCC amino-proximal zinc finger domain, which has been demonstrated to suppress the host’s antiviral immune response [7,8]. This suppression occurs via the hyperphosphorylation of phenylalanine/glycine-rich nuclear pore proteins, facilitated by the binding of the L protein to Ran-GTPase, thereby disrupting nuclear-cytoplasmic transport [9]. The 2A protein plays a critical role in –1 ribosomal frameshifting by forming an RNA–protein complex with a downstream stem-loop structure, which regulates the expression of viral genes. Notably, the partial removal of the 2A protein from the EMCV genome disrupts the processing of capsid individual proteins [10,11]. One of its key functions is to interact with annexin A2, a protein that is involved in various cellular processes, including apoptosis and stress responses [12]. L protein is recognized for its significant role in the inhibition of cellular translation, although the precise mechanisms underlying this inhibition remain unclear [13]. Evidence indicates that the suppression of L protein function leads to a decrease in the efficiency of viral translation. Additionally, the impairment of both security proteins of EMCV has been linked to the induction of caspase-dependent apoptosis in infected HeLa cells [14].

One of such cytoprotective pathways, activated during viral infection and the rapid production of viral proteins, is unfolded protein response, a cellular stress response that controls the levels of proteins with wrong structures in the endoplasmic reticulum [15]. This response may activate a subset of mechanisms to restore cell homeostasis and promote cell viability. However, it may also lead to programmed cell death [16,17]. The interaction between viral infections and UPR plays a significant role in determining the cell’s fate.

Three primary UPR sensors are activated during ER stress: inositol-requiring enzyme 1 (IRE1), PKR-like endoplasmic reticulum kinase (PERK), and activating transcription factor 6 (ATF6) [15,18,19]. The IRE1-XBP1 branch is one of the conserved mechanisms of the unfolded protein response [20]. IRE1 detects ER stress through its luminal domain [21]. Upon activation, IRE1 undergoes oligomerization and autophosphorylation, leading to the unconventional splicing of X-box binding protein 1 (XBP1) mRNA, producing the transcription factor XBP1s. XBP1s activate the expression of genes involved in protein folding, ER-associated degradation (ERAD), and lipid biosynthesis [21,22,23]. Additionally, IRE1’s endoribonuclease activity can target and degrade specific mRNAs through a mechanism called IRE1-dependent decay (RIDD), reducing the burden on the ER [24,25]. Beyond these functions, IRE1 signaling can also influence cell fate decisions, with prolonged ER stress leading to apoptosis if homeostasis cannot be restored [26]. Thus, IRE1’s activity is intricately linked to either cell survival or death under ER stress conditions.

Previously, UPR activation was described for different representatives of the *Picornaviridae* family [27,28,29]. Additionally, EMCV has been found to induce autophagy by activating the UPR in BHK-21 cells. In these cells, the levels of Grp78 and Grp94 proteins were elevated, and the ATF6 pathway was activated when expressing 2C or 3D non-structural proteins or upon infection with EMCV. However, the authors observed no significant changes in XBP1 mRNA splicing and did not explore the levels of IRE1 protein in their research [30]. Furthermore, it has been demonstrated that the HSP90 protein influences the replication of EMCV, suggesting a complex interplay between UPR activation and EMCV infection [31].

This paper explores the modulation of the unfolded protein response during encephalomyocarditis virus infection in HeLa cells, with a specific focus on the IRE1-XBP1 pathway. We examined changes in phosphorylated IRE1 levels and the activity of XBP1 splicing in infected cells.

## 2. Materials and Methods

### 2.1. Cells and Viruses

HeLa cells were grown in Dulbecco’s modified Eagle’s medium (DMEM, FSBSI “Chumakov FSC R&D IBP RAS, Russia”) with 10% fetal bovine serum (Gibco, Billings, MT, USA) at 37 °C in a 5% CO_2_ humidified atmosphere. Infection was performed in 35 mm plates. Cells were infected with either virus type (EMCV wt, EMCV_ΔL, EMCV_Δ2A, or EMCV_ΔL_Δ2A) at an MOI of 40 PFU/cell after two washes with serum-free media. Incubation was performed at room temperature for 30 min. The wild-type Mengo strain of EMCV serotype 1 (WT) was derived from the plasmid pM16.1. The construction of virus mutants based on the EMCV wild-type scaffold was described earlier [9]. EMCV_ΔL had the substitutions Cys19 → Ala and Cys22 → Ala in the L protein sequence, EMCV_ Δ2A involved a deletion spanning amino acids 11 to 125 in the 2A protein, and EMCV_ΔL_Δ2A incorporated both of these modifications. Mock-infected HeLa cells were used as a negative control in all experiments. HeLa cells treated with DTT (Sigma Aldrich, Saint Louis, MO, USA, #D0632, up to 10 mM) were used as the positive control. All the experiments were performed in a containment environment.

### 2.2. Western Blot Analysis of HeLa Cell Lysates

At the specified time points, either virus- or mock-infected cells were lysed in Laemmli buffer (2% SDS, 50 mM 2-mercaptoethanol, 50 mM Tris-HCl [pH 6.8]). Samples were separated using gradient SDS-PAGE (6–12%) and transferred onto a nitrocellulose membrane (Bio-Rad, #1620115). The membranes were then blocked in 5% non-fat dry milk in TBS (114 mM NaCl, 17 mM Tris-HCl [pH 8.0]) with 0.05% Tween 20 for 1 h and incubated overnight at 4 °C with primary antibodies (diluted 1:1000 to 1:2000). Antibodies against p-IRE1 (Abcam, Waltham, MA, USA, #ab124945; Affinity Biosciences, #DF8322) were used to measure phosphorylated IRE1 levels. After washing the membranes, they were incubated with the appropriate HRP-conjugated secondary antibody for 1 h at room temperature and visualized using Clarity ECL substrate (Bio-Rad, Hercules, CA, USA). For loading control, the blots were treated with β-actin antibodies (Sigma, Saint Louis, MO, USA, #A5316).

### 2.3. RNA Extraction and RT-qPCR

Total RNA was extracted from cells using the ExtractRNA reagent (#BC032, Evrogen, Moscow, Russia) following the manufacturer’s protocol. The RNA samples were treated with DNase I to avoid genomic DNA contamination. The cDNA was synthesized using M-MLV reverse transcriptase (Invitrogen, Waltham, MA, USA) and random hexamer primers (Evrogen, Moscow, Russia). RT-qPCR was performed with the following primer sets: for the XBP1 spliced mRNA, forward: 5′-aatgaagtgaggccagtggc-3′, reverse: 5′-tgaagagtcaataccgccagaa-3′, probe: 5′-(FAM)tgctgagtccgcagcaggtgca(RTQ1)-3′. The values were normalized to the level of the RPL19 transcript obtained with the following primers: forward: 5′-agcggattctcatggaaca-3′, reverse: 5′-ctggtcagccaggagctt-3′, probe: 5′-(FAM)tccacaagctgaaggcagacaagg(RTQ1)-3′. The mock-infected cells treated with 10 mM DTT for 2 h were used as a positive control for UPR activation. The PCR reactions were set up as follows: 5 min at 95 °C, followed by 40 cycles of 20 s at 95 °C and 40 s at 60 °C. The R-412 qPCR kit (Syntol, Moscow, Russia) was used. The data were analyzed with QuantStudio 5 Software (Thermo Fisher Scientific, Waltham, MA, USA).

### 2.4. PCR Experiment for Differentiation Spliced and Unspliced XBP1 mRNA Forms

For the PCR experiment followed by agarose gel electrophoresis, the primers 5′-ccttgtagttgagaaccagg-3′ and 5′-ggggcttggtatatatgtgg-3′ were used, producing fragments of either 442 or 416 bp (specific for the unspliced or spliced XBP1 mRNAs, respectively). In this case, the PCR reactions were set up as follows: 5 min at 95 °C, followed by 40 cycles of 30 s at 95 °C, 30 s at 58 °C, 60 s at 72 °C, and then 7 min at 72 °C.

### 2.5. Western Blot Quantification Analysis

The luminescence signal was recorded using the ChemiScope Gel Documentation System. Western blot data were quantified using ImageJ software version 1.53k. Statistical comparisons between the two datasets were performed using Student’s *t*-test with Prism 8 software. A *p*-value of less than 0.05 was considered statistically significant.

## 3. Results

### 3.1. IRE1 Phosphorylation Is Activated in EMCV-Infected HeLa Cells

**Figure 1:**
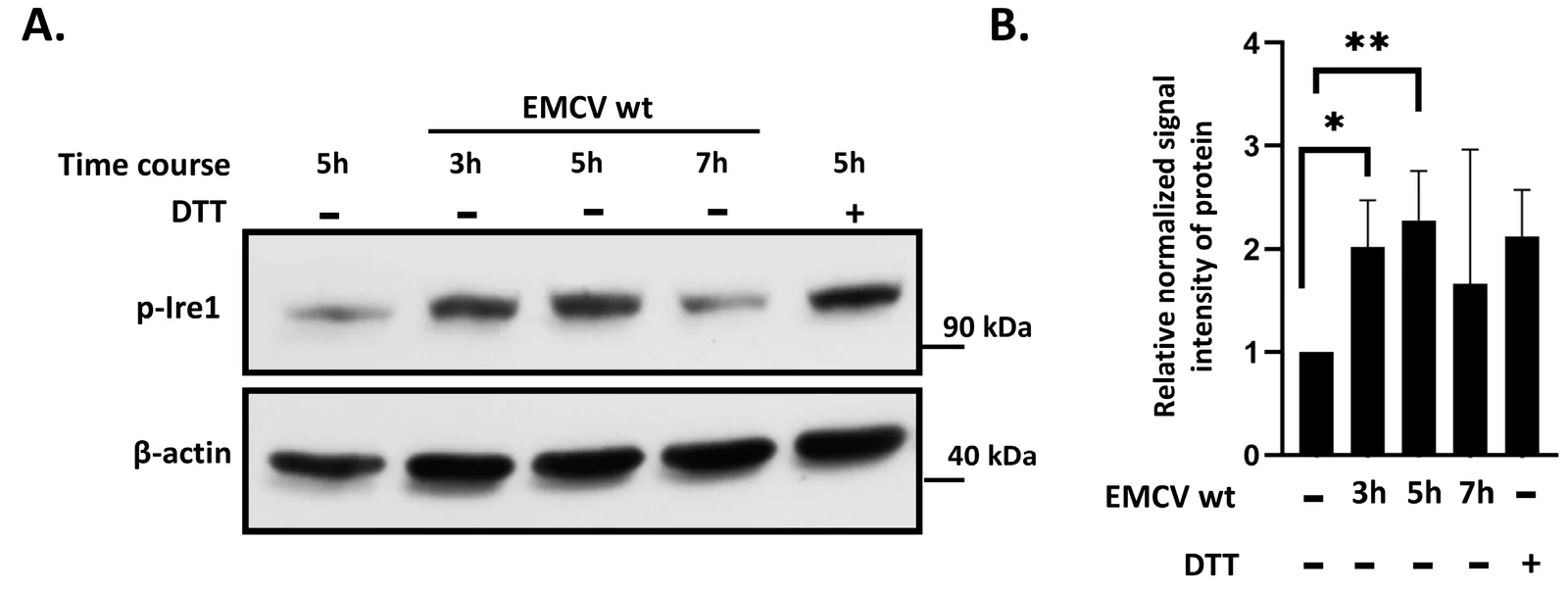
IRE1 phosphorylation in EMCV (Mengo strain)-infected HeLa cells. (**A**) Phosphorylated IRE1 and total β-actin levels in HeLa cells infected with EMCV Mengo strain. (**B**) Quantified normalized signal intensity of phosphorylated IRE1 to β-actin, relative to mock-infected cells. Representative results of at least three independent experiments are shown. * *p* < 0.05, ** *p* < 0.01.

To determine whether EMCV infection activates the IRE1-mediated pathway of the unfolded protein response (UPR), we examined the levels of phosphorylated IRE1 protein in infected cells. HeLa cells were infected with EMCV at an MOI of 40 pfu/cell, and protein lysates were collected at 3, 5, and 7 h post-infection. Cells treated with DTT (10 mM), a strong ER stress inducer, were used as a positive control (Figure 1). Phosphorylated IRE1 forms were detected as early as 3 h post-infection in EMCV-infected cells, compared to mock-infected cells, as shown by immunoblotting with monoclonal antibodies against phospho-IRE1. However, as the infection progressed, we observed a subsequent downregulation of phosphorylated IRE1 protein. By 7 h post-infection, the levels of phospho-IRE1 were comparable to those in mock-infected cells.

### 3.2. IRE1 Phosphorylation Levels in HeLa Cells Infected with EMCV Deficient in L or 2A Protein

**Figure 2:**
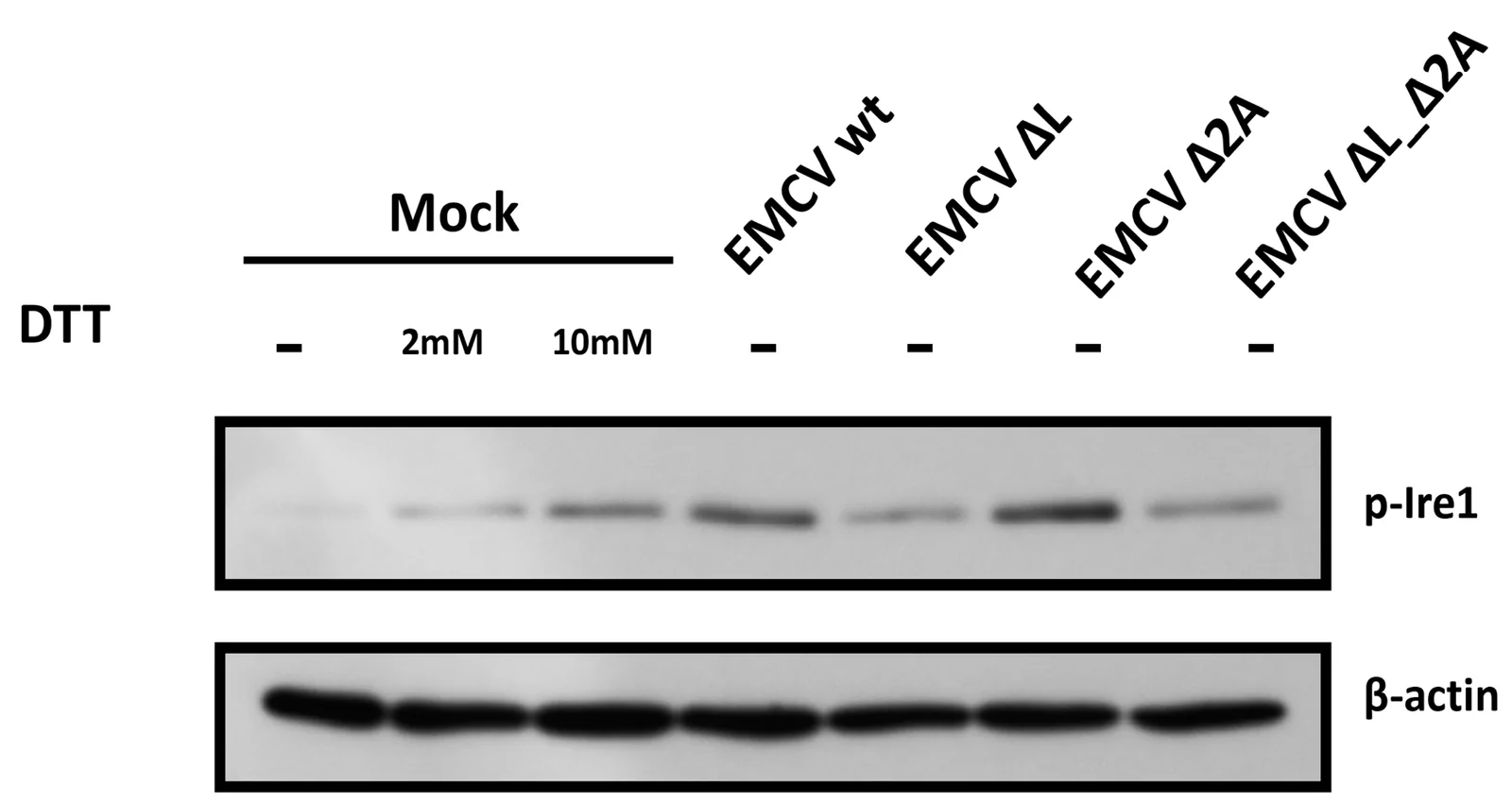
IRE1 phosphorylation in EMCV (Mengo strain) and its mutant infected HeLa cells.

We investigated the role of the EMCV “security” proteins, L and 2A, in IRE1 phosphorylation by comparing the levels of phosphorylated IRE1 in HeLa cells infected with four EMCV variants: wild-type and viruses lacking either the L protein, the 2A protein, or both protein functions. EMCV variants with each of these mutations were previously generated in our laboratory [14]. Hela cells were infected with either of these viruses at an MOI of 40 pfu/cell. The IRE1 phosphorylation level in infected cells was analyzed by immunoblot and compared to mock-infected cells as well as Hela cells treated with DTT- 2 mM or 10 mM, respectively (Figure 2).

At 5 h post-infection, we observed varying levels of IRE1 phosphorylation in HeLa cells infected with either wild-type EMCV or its variants lacking specific “security” proteins (Figure 2). Notably, cells infected with EMCV_ΔL or EMCV_ΔL_Δ2A exhibited slightly reduced levels of phosphorylated IRE1 compared to those infected with EMCV_wt and EMCV_2A.

Previous studies have demonstrated that the functional inactivation of the L protein, but not 2A, results in diminished viral genome replication during EMCV infection in HeLa cells [13]. The observed changes in IRE1 phosphorylation levels in cells infected with these EMCV variants may stem from replication deficiencies or could reflect an indirect effect of the L protein on IRE1 phosphorylation. This hypothesis warrants further investigation to confirm the underlying mechanisms.

### 3.3. Inhibition of XBP1 mRNA Splicing in EMCV-Infected HeLa Cells

To assess the effect of EMCV infection on XBP1 mRNA splicing, we performed a quantitative real-time PCR on HeLa cells infected with wild-type EMCV and its variants lacking the functions of the L or 2A proteins, either individually or simultaneously. The levels of spliced XBP1 mRNA were measured both under normal conditions and after treatment with DTT, an inducer of ER stress.

**Figure 3:**
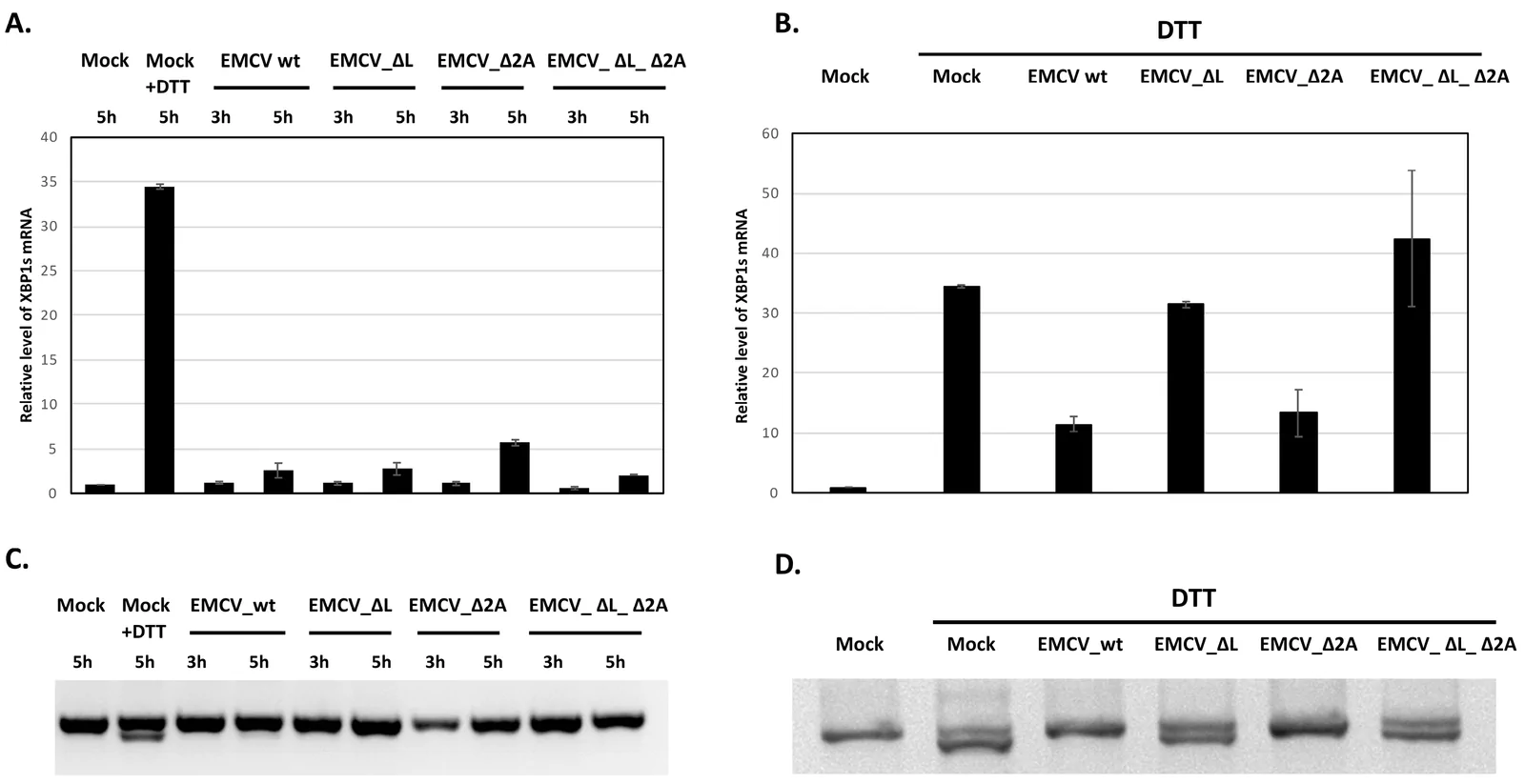
EMCV modulates XBP1 mRNA splicing in infected HeLa cells. (**A**) Total RNA from HeLa cells infected with wild-type EMCV and its mutants; mock-infected; and treated with 10 mM DTT was isolated at the indicated time points. The relative level of the spliced Xbp1 mRNA was measured by RT-qPCR with primers specific to Xbp1s mRNA isoform and to RPL19 mRNA as a reference. (**B**) HeLa cells were infected with EMCV and its mutants, then at 3 hpi, 10 mM DTT was added to the medium. Two hours later, total RNA was isolated and the relative levels of the spliced Xbp1 mRNA were measured by RT-qPCR. (**C**) The same experiment as in (**A**), RT-PCR with an Xbp1-specific primer pair producing fragments of either 442 or 416 bp (corresponding to the unspliced or spliced Xbp1 mRNAs, respectively), was performed, and fragments were analyzed on 2% agarose gel. (**D**) The same experiment as in (**B**), where fragments corresponding to unspliced and spliced form of XBP1 mRNA were analyzed on 2% agarose gel, was performed.

Under normal conditions, the levels of spliced XBP1 mRNA in cells infected with all EMCV variants, including wild-type EMCV and the L- and 2A-deficient mutants, were significantly lower compared to mock-infected cells treated with an ER stress inducer (Figure 3A). We also performed 2% agarose gel electrophoresis to differentiate RT-PCR products corresponding to spliced and unspliced XBP1 mRNA isoforms in cells infected with wild-type EMCV and its mutants lacking either one or both “security” proteins. The spliced variant was not detected in cells infected with EMCV or its mutants, whereas it was detected in HeLa cells treated with DTT (Figure 3C).

This may lead to a hypothesis that EMCV infection suppresses XBP1 mRNA splicing in the absence of chemical stress induction, regardless of the specific roles of the L and 2A proteins. Following treatment with DTT (10 mM), a significant increase in the levels of spliced XBP1 mRNA was observed in mock-infected cells, with a 30-fold rise in expression relative to untreated cells. In contrast, cells infected with wild-type EMCV showed a marked suppression of splicing, with the relative expression of spliced XBP1 mRNA being approximately three times lower than that in mock-infected cells (Figure 3B). Interestingly, this suppression was not observed in cells infected with the L-deficient EMCV variant, where DTT treatment resulted in levels of spliced XBP1 mRNA comparable to those in mock-infected cells. However, cells infected with the EMCV variant deficient in 2A protein continued to exhibit the suppression of XBP1 mRNA splicing, similar to wild-type EMCV infection. Our results were supported by analyzing RT-PCR products of unspliced and spliced XBP1 mRNA isoforms on 2% agarose gel electrophoresis (Figure 3D).

The observed impact of the L-“security” protein on the suppression of XBP1 mRNA splicing may stem from either the replication deficiencies of the corresponding EMCV variants in HeLa cells or the involvement of this security protein in the molecular mechanisms underlying this process [14].

## 4. Discussion

Encephalomyocarditis virus (EMCV), a member of the *Picornaviridae* family, is known for its ability to manipulate cellular mechanisms [5,6,13,32].

In this study, we observed that encephalomyocarditis virus (EMCV) triggers the autophosphorylation of the IRE1 protein kinase/endoribonuclease in HeLa cells during the early mid-infection phase, as demonstrated by immunoblotting with specific antibodies against the phosphorylated form of IRE1 in various experiments (Figure 1). The additional information and data are shown in the Supplementary Materials provided (Figure S1 and Figure S2). However, this phosphorylation does not result in the accumulation of spliced XBP1 mRNA. Our findings suggest that EMCV’s influence on IRE1 may disrupt the typical ER stress response without promoting the expected downstream signaling.

Our data indicate that, similar to poliovirus, EMCV can suppress the IRE1-dependent protein folding pathway by inhibiting XBP1 mRNA splicing, thereby preventing a proper cellular response to ER stress [33]. This suppression, presumably mediated by viral proteins, may enable the virus to evade host defense mechanisms and enhance viral survival in particular cell types.

Although both poliovirus and EMCV suppress host defense mechanisms through similar pathways, our findings highlight differences in their mechanisms of action. In the 2022 study, it was hypothesized that poliovirus suppresses UPR by inhibiting IRE1 activation (by proteolysis). In EMCV-infected HeLa cells, IRE1 proteolysis was not observed [33].

Furthermore, EMCV’s L protein may possess additional functions that might mediate the suppression of XBP1 splicing under ER stress conditions. We found that, in the absence of chemical stressors, XBP1 mRNA splicing was suppressed across all EMCV variants, including those deficient in L or 2A proteins (Figure 3A,C). Our data align with previous findings by Hou et al. [30] which demonstrate that EMCV infection does not result in XBP1 mRNA splicing in BHK-21 cells. However, following DTT treatment, significant differences emerged in cells infected with L-deficient EMCV that did not suppress XBP1 mRNA splicing, whereas 2A-deficient EMCV maintained the ability to suppress splicing (Figure 3B,D). This is the first demonstration of XBP1 mRNA splicing inhibition in EMCV-infected cells.

Our results also indicate that infection with L-deficient EMCV mutants not only leads to a reduced suppression of Xbp1 mRNA splicing but may also be associated with lower levels of phosphorylated IRE1 compared to cells infected with wild-type EMCV (EMCV_wt) and the EMCV_2A variant (Figure 2, Figure S3, and Figure S4).

However, these differences may stem from a reduced capacity for viral genome replication during EMCV infection in HeLa cells [14]. Ivin et al. showed that inactivating a single security leader protein in EMCV leads to a fivefold decrease in viral yield in HeLa cells between 4 and 8 h post-infection (h.p.i.), as well as the reduced expression of viral proteins.

Previous studies have shown that the L protein can indirectly influence the phosphorylation status of cellular proteins, thereby affecting their function [5,6]. A similar mechanism may also be relevant in this context, as we observed distinct phosphorylation profiles of IRE1 in cells infected with EMCV lacking L or 2A function, along with corresponding effects on XBP1 mRNA splicing. However, further experiments are needed to confirm this hypothesis. Overall, our findings highlight the complexity of viral–host interactions and underscore the importance of investigating multiple viral components to fully understand their contributions to pathogenesis.

Another intriguing observation is that the inhibition of XBP1 mRNA occurs in the context of IRE1 phosphorylation, which is unexpected since such phosphorylation typically activates the RNase activity of IRE1. We propose that an alternative RNase activity of IRE1, known as regulated IRE1-dependent decay (RIDD), may be involved. Evidence suggests that host RIDD activity on BLOS1 enhances the intracellular replication of coronaviruses [34]. Moreover, the inhibition of the RIDD pathway led to a decrease in the steady-state levels of several viral proteins and a significant reduction in the JEV (Japanese encephalitis virus) titer in the supernatant of infected cells [35]. This could provide a path for a further investigation into the modulation of the IRE1-XBP1 pathway in EMCV-infected cells

Further research should focus on understanding EMCV’s interaction with cellular UPR mechanisms in greater detail. Specifically, attention should be given to the differences in viral protein activity in different cell lines, as well as the role of other viral factors that may assist in suppressing the cellular ER stress response. We believe that investigating these mechanisms could support the development of antiviral strategies aimed at restoring proper UPR function.

## 5. Conclusions

Our study demonstrates that EMCV infection activates IRE1 phosphorylation in HeLa cells, indicating an unfolded protein response (UPR). The inhibition of XBP1 mRNA splicing suggests that the virus can manipulate these host responses to its advantage. This modulation may be driven by viral proteins that interact with host factors, disrupting normal cellular processes.

## Data Availability

The original contributions presented in this study are included in the article/supplementary material. Further inquiries can be directed to the corresponding author.

## Supplementary Materials

The following supporting information can be downloaded at: https://www.mdpi.com/article/10.3390/v17030360/s1, Figure S1: IRE1 phosphorylation in EMCV-infected HeLa cells (another representative result in addition to that shown in Figure 1). Phosphorylated Ire1 was detected at 3 h post infection with subsequent downregulation; Figure S2: IRE1 phosphorylation in EMCV-infected HeLa cells (another representative result in addition to that shown in Figure 1, with different time points). Maximal level of phosphorylated IRE1 was detected at 4 h post infection; Figure S3: IRE1 phosphorylation in EMCV (Mengo strain) and its mutants infected HeLa cells (another representative result in addition to that shown in Figure 2); Figure S4: IRE1 phosphorylation in EMCV (Mengo strain) and its mutants infected HeLa cells (another representative result in addition to that shown in Figure 2).
