# Supplementary material for: Modulation of Ire1-Xbp1 Defense Pathway in Encephalomyocarditis Virus-Infected HeLa Cells
Source: Viruses. 2025 Mar 2;17(3):360. doi: 10.3390/v17030360 (PMC11946305; doi:10.3390/v17030360)
Supplement: Supplementary file 1 [file viruses-17-00360-s001.zip › viruses-3470034-supplementary.pdf]

Communication

Modulation of Ire1-Xbp1 defense pathway in EMCV-infected HeLa cells

Anna Shishova [1,2]*, Yury Ivin [1], Ekaterina Gladneva [1], Ksenia Fominykh [1], Ilya Dyugay [1] and Anatoly Gmyl [1†].

Supplementary materials

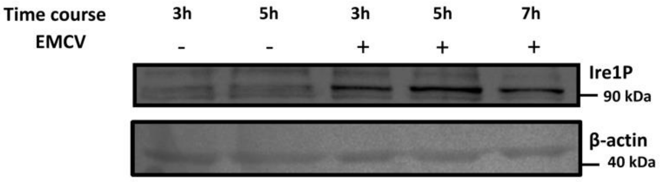

Figure S1. IRE1 phosphorylation in EMCV-infected HeLa cells (another representative result in addition to that shown in Figure 1). Phosphorylated Ire1 was detected at 3 hours post infection with subsequent downregulation.

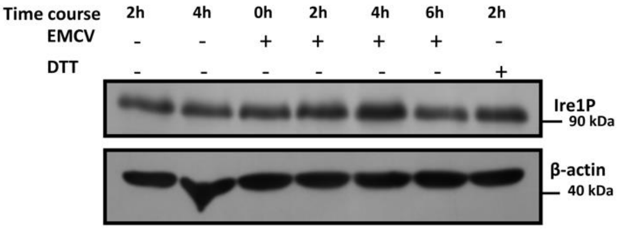

Figure S2. IRE1 phosphorylation in EMCV-infected HeLa cells (another representative result in addition to that shown in Figure 1, with different time points). Maximal level of phosphorylated IRE1 was detected at 4 hours post infection.

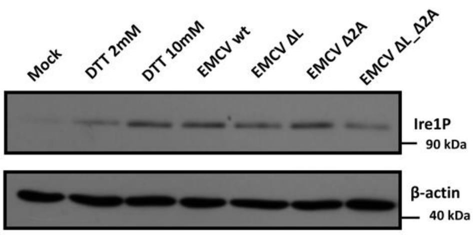

Figure S3. IRE1 phosphorylation in EMCV (Mengo strain) and its mutants infected HeLa cells (another representative result in addition to that shown in Figure 2).

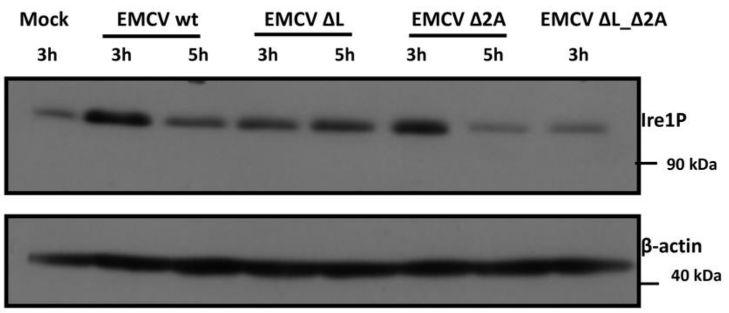

Figure S4. IRE1 phosphorylation in EMCV (Mengo strain) and its mutants infected HeLa cells (another representative result in addition to that shown in Figure 2).
